# Synthesis of IR-emitting HgTe quantum dots using an ionic liquid-based tellurium precursor†

**DOI:** 10.1039/d1na00291k

**Published:** 2021-06-28

**Authors:** Hassan Mirzi, Simon M. Fairclough, Richard J. Curry, Sarah J. Haigh, Mark Green

**Affiliations:** Department of Physics, King's College London The Strand London WC2R 2LS UK mark.a.green@kcl.ac.uk; Department of Electrical and Electronic Engineering, The Photon Science Institute, University of Manchester Oxford Road Manchester M1 3BB UK; Department of Materials, University of Manchester Oxford Road Manchester M13 9PL UK

## Abstract

New scalable precursor chemistries for quantum dots are highly desirable and ionic liquids are viewed as an attractive alternative to existing solvents, as they are often considered green and recyclable. Here we report the synthesis of HgTe quantum dots with emission in the near-IR region using a phosphonium based ionic liquid, and without standard phosphine capping agents.

## Abstract 2

In this communication, we highlight the use of an ionic liquid-based tellurium precursor in the synthesis of HgTe quantum dots.

## Graphical abstract

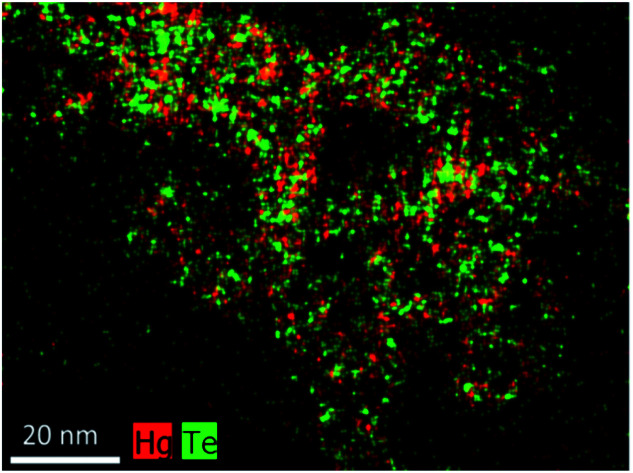

Solution phase synthesis routes to high-quality semiconductor nanoparticles have been growing in demand due to the increase in the applications of these materials within the field of fluorescent labelling, photovoltaics and light-emitting devices.^1^ HgTe quantum dots are the most popular type of mercury chalcogenide nanoparticles with vast potential for applications in the optoelectronics industry.^2,3^ Initial synthetic strategies for HgTe nanocrystals highlighted the rapid low temperature growth of the particles after nucleation due to the highly reactive nature of the mercury precursor.^4,5^ In our previous work on HgSe quantum dots (QDs) we used liquid nitrogen to separate the nucleation and particle growth during synthesis, resulting in a narrow size distribution with improved optical properties. Here we have adapted and applied this process towards synthesising HgTe QDs.

Synthesising high-quality telluride nanocrystals requires the preparation of a soluble tellurium precursor.^6^ Trioctylphosphine (TOP) has commonly been used to achieve this, hence the majority of HgTe synthesis reactions consist of a TOP-Te precursor combining with a Hg precursor, normally a salt, in the presence of long chain Lewis base.^2^ In the past decade, work has been carried out to synthesise semiconductor nanocrystals without the use of phosphine based passivating agents. Initially this was because such ligands have proved deleterious in biological applications^7^ but more recently, alternative ligands have been utilised during the synthesis of HgTe quantum dots to avoid the production of aggregated particles.^8^

Previous reports describe the synthesis of HgSe nanoparticles without the use of TOP, utilising selenium in octadecene.^9^ Nonetheless the metallicity of tellurium is much stronger than Se, presenting a challenge for creating a stable Te precursor. In the past few years, ionic liquids have been used for preparing a variety of nanoparticles such as aluminum antimonide,^10^ metal fluorides^11^ and cobalt platinum alloys.^12^ Certain ionic liquids exhibit desirable similarities with existing capping agents, such as commercial availability, thermal stability at elevated temperatures, long alkyl chains, and functional groups allowing coordination with the particle's surface. We have previously reported the use of a phosphonium ionic liquid in the preparation of luminescent CdSe quantum dots, however, the use of the same system to prepare telluride quantum dots is inaccessible as elemental tellurium does not appear to be readily soluble in the solvent. Thus, modifications needed to be made to the previous method as a means to support a stable reaction.

Inspired by a report from Wei *et al.*,^13^ elemental tellurium was reduced by NaBH_4_ in trihexyltetradecylphosphonium bis(2,4,4-trimethylpentyl)phosphinate upon heating, giving a viable solution precursor.^14^ In a typical synthesis, mercury acetate was added to oleic acid under an inert atmosphere and heated to 90 °C. The solution was then stabilised at that temperature and exposed to vacuum for 2 hours. In a separate flask, the ionic liquid, octadecene and sodium borohydride were heated under an inert atmosphere to 220 °C for *ca.* 1 hour, forming a purple-coloured solution indicating a successful reaction. Both solutions were then left to cool to room temperature whilst maintaining the inert atmosphere. The tellurium solution was then injected into the mercury solution in a single, rapid injection under an inert atmosphere with continuous stirring. Following the injection, the flask was immediately immersed in liquid nitrogen, freezing the reaction solution. The reaction was allowed to warm to room temperature over a matter of hours, with stirring, yielding a black solution. The resulting quantum dots were then isolated using acetone and toluene as a non-solvent/solvent pair and purified by size selective precipitation.

Low resolution transmission electron microscope (TEM) images (ESI Fig. 1†) showed relatively monodispersed particles with an average particle diameter of 5 nm ± 0.7 nm. The high-resolution transmission electron microscope images (HRTEM) in Fig. 1 show the nanocrystals with a clearly aggregated, extended morphology, with visible lattice planes suggesting twinned crystals and tetrahedral facets, consistent with a zinc blende structure. Scanning transmission electron microscope (STEM) and energy dispersive X-ray analysis (EDX) imaging (ESI Fig. 2†) confirmed the elemental composition of Hg and Te to be *ca.* 48.6% and *ca.* 51.4% respectively, as expected for HgTe. The measured XRD pattern shown in Fig. 2 confirms the HgTe crystalline formation with three broad diffraction peaks corresponding with 111, 220 and 311 planes of the zinc-blend phase of HgTe.

**Fig. 1:**
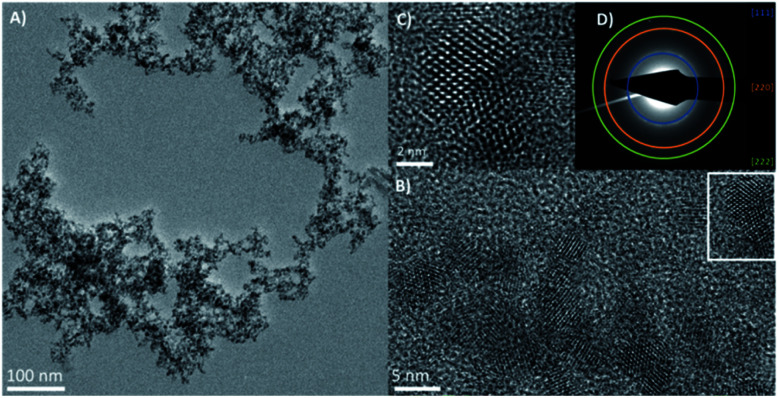
High resolution TEM images of HgTe samples synthesised at room temperature with injection/freezing – (A) overview TEM image of QD aggregates; (B and C) HRTEM images of QD showing highly crystalline particles (C) is an enlargement of the region highlighted in (B) showing twinning faults in the crystal structure. (D) Selected area electron diffraction (SAED) pattern confirming the zinc blende HgTe structure.

**Fig. 2:**
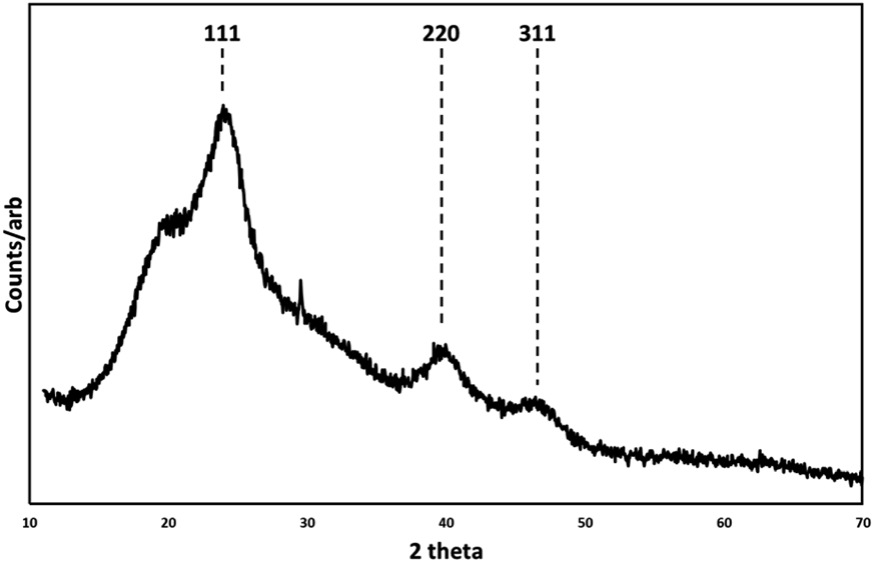
X-ray diffraction pattern (XRD) of zinc blende HgTe particles prepared at room temperature and associated Miller indices. Background feature at 18 degrees 2*θ*.

The aggregated particle structure seen in Fig. 1 is similar to that observed for ionic liquid passivated CdSe.^14^ Whilst monodisperse isolated nanoparticles are generally preferred for most applications using quantum dots, in the case of photodetectors and IR devices, nanoparticle aggregation is encouraged as it provides natural electronic coupling between the particles, reducing the barrier width between them.^15^ For these applications, ligand exchange within a quantum dot film is usually essential for achieving conductivity between the drop cast particles.^16^ For example, capping with HgTe nanoparticles with shorter dithiol molecules, such as ethanedithiol (EDT), using solid-state ligand exchange, has been shown to improve photoconductivity between the particles without significantly compromising their colloidal stability.^17^ Only when aggregated have HgTe quantum dots been shown to be readily conductive without the need for any further treatment.^15^ It is assumed that the ionic liquid coordinates to the particle surface in a similar manner to previous reports on ionic liquids passivated CdSe quantum dots.^14^

The optical data for the as-prepared HgTe quantum dots are shown in Fig. 3 (solid line for absorption, dotted line for emission). The absorption spectrum exhibited a clear excitonic shoulder at *ca.* 800 nm and a gradual onset of absorption at *ca.* 1100 nm, consistent with numerous other reports for HgTe.^2,15–17^ Band edge emission for such quantum dots was observed with a maxima. at *ca.* 1150 nm, and a slight shoulder at *ca.* 1275 nm.

**Fig. 3:**
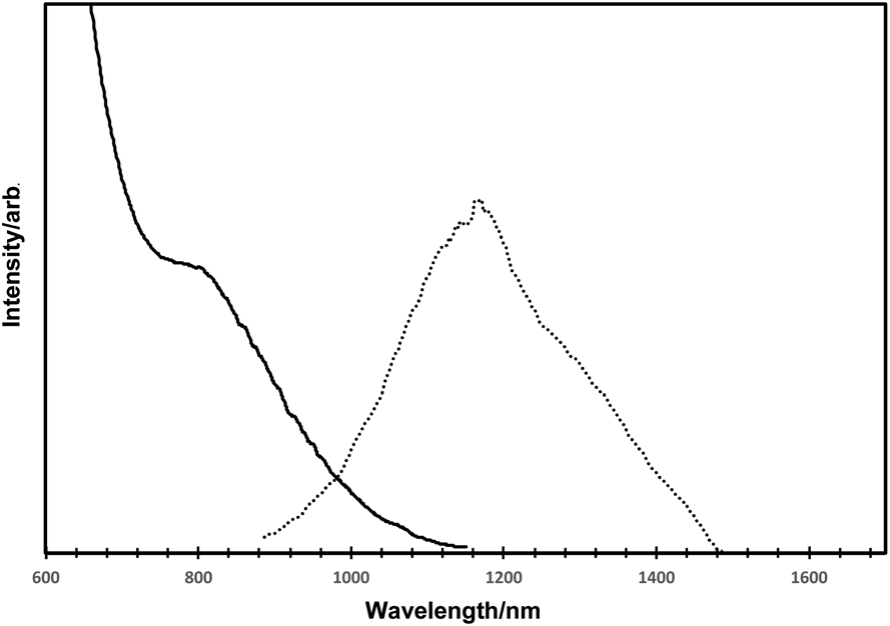
Absorption spectra (solid lines) and emission spectra (dotted lines) of HgTe quantum dots.

The emission quantum yield was calculated using the integrating sphere method and was approximately 9%.

In conclusion, we have developed a phosphine free-route to IR-luminescent HgTe quantum dots using an ionic liquid-based tellurium precursor, with a rapid liquid nitrogen freezing step to separate particle nucleation and growth. Following size fractionation, distinct optical states could be observed in the absorption spectra, accompanied with band edge emission.

## Conflicts of interest

There are no conflicts to declare.

## Supplementary Material

NA-003-D1NA00291K-s001
