# Supplementary material for: Synthesis of IR-emitting HgTe quantum dots using an ionic liquid-based tellurium precursor
Source: Nanoscale Adv. 2021 Jun 28;3(14):4062–4. doi: 10.1039/d1na00291k (PMC9418010; doi:10.1039/d1na00291k)
Supplement: NA-003-D1NA00291K-s001 [file NA-003-D1NA00291K-s001.pdf]

Electronic Supplementary Material (ESI) for Nanoscale Advances.
This journal is © The Royal Society of Chemistry 2021

Synthesis of IR emitting HgTe quantum dots using an ionic liquid-based tellurium precursor.

Hassan Mirzi, Simon M. Fairclough, Richard J. Curry, Sarah J. Haigh, Mark Green.

Supporting information

Chemicals

All reagents and materials were obtained from commercial sources and used as received unless otherwise noted. The following are all chemicals used in the reaction procedures:

Mercury(II) acetate (97 %, Sigma-Aldrich), tellurium powder (99.8 %, Sigma-Aldrich), sodium borohydride (99%, Sigma-Aldrich), technical grade oleic acid (90 %, Sigma-Aldrich), Tetradecyltrihexylphosphonium bis(2,4,4-trimethylpentyl)phosphinate (90%, Sigma-Aldrich), technical grade 1-octadecene (90 %, Sigma-Aldrich), toluene (BDH), acetone (BDH), liquid nitrogen (KCL labs), deionised water (18.2 MΩ, KCL labs).

Preparation of solvents and air sensitive compounds

Solvents for use with air stable chemicals were used as received. Toluene was refluxed over sodium for 1 hour under nitrogen atmosphere and then distilled. Octadecene was degassed inside a two-neck Schlenk flask under vacuum at 100 °C for one hour and kept under nitrogen atmosphere before being used. Oleic acid was stored inside a conventional fridge to prevent oxidation. Air-sensitive reagents were handled under nitrogen. Reactions were carried out under nitrogen atmosphere using a Schlenk line consisting of four vacuum and nitrogen arms. Schlenk type glassware was used when connected to the manifolds using rubber pressure tubing. The nitrogen or vacuum conditions were selected using a valve which either allowed nitrogen or vacuum suction through the tubes. A rotary oil pump maintained the vacuum with nitrogen being supplied by a cylinder connected directly to the nitrogen manifold. A fume cupboard was used to manipulate and measure solid chemicals in suitable glassware. Liquid air-sensitive chemicals were delivered by cannula or by injection from glass syringes to Schlenk flasks securely fitted with rubber septa.

Purified colloidal nanoparticles in aqueous solution were stored in vials inside a conventional fridge for immediate testing or kept inside a fume cupboard under nitrogen atmosphere for future use.

Particle synthesis

Mercury(II) acetate (0.44 g, 1.4×10^{-3} moles) was dissolved in 20 ml of oleic acid (OA) inside a 100ml two-neck Schlenk flask and heated to approximately 90 °C whilst stirring to affect dissolution, then cooled to room

temperature. Experiments were also performed by keeping the mercury (II) acetate and OA mixture under vacuum for an extended period, in this case two hours, to remove any excess build-up of acetic acid known to cause aggregation of the nanoparticles.

The Te precursor was prepared inside a 100ml two-neck Schlenk flask by adding tellurium power (0.1915 g, 1.5 × 10^{-3} moles) and sodium borohydride (0.0598 g, 1.6 × 10^{-3} moles) to 20 ml of purified degassed octadecene and 11ml of tetradecyltrihexylphosphonium bis(2,4,4-trimethylpentyl)phosphinate ionic liquid (0.1 M solution). The mixture was stirred and heated to 220 °C for approximately 1 hour to fully dissolve the tellurium powder, before being cooled to room temperature. The solution turned a dark purple colour when heated to 220 °C and stayed purple after cooling to room temperature. It is worth noting that exposure to air for more than 1 min turned the precursor solution dark green, causing Te to precipitate out of the solution. When this happened, the precursor solution was vacuumed for another hour and reheated to 220 °C under nitrogen. The temperature was monitored using a digital thermometer inserted inside the flask through a rubber septum.

In a typical HgTe reaction, the preparation of the Hg and OA precursor was followed by injection of 13 ml of the 0.1 M Te precursor solution (1.3 × 10^{-3} moles) using a glass syringe. The injection was carried out at room temperature under nitrogen atmosphere, followed by immediate freezing in liquid nitrogen using a low form glass dewar and gradual warming to room temperature. After growth was completed, the resulting solution colour was found to be black/dark brown after ca. 5 hours.

After particle growth, acetone was added to the solution, followed by centrifugation for 10 minutes resulting in the precipitation of a black solid. Acetone was used as it is a more polar solvent than methanol and increasing the overall polarity of the solution caused larger particles/aggregates to precipitate out as they are less stable. By adding toluene, followed by centrifuging for a further 2 minutes and filtration, a black solution was obtained; containing the nanoparticles dispersed in the organic solvent. It is worth noting that the HgTe samples precipitated after only one week.

Transmission electron microscopy (TEM, HRTEM)

Transmission electron microscope (TEM) images for the colloidal HgTe nanoparticles were obtained using a FEI TECNAI 20 microscope at 200 kV at the Centre for Ultrastructural Imaging, KCL. The HRTEM for the chemically synthesised colloidal HgTe nanoparticles were collected at University of Manchester, Department of Materials using a probe corrected FEI Titan G2 80–200 (S)TEM system at 200 kV. All TEM samples were prepared by dropping a very low concentration toluene distributed nanoparticle solution on to a carbon film TEM grid.

Scanning transmission electron microscopy (STEM)

STEM imaging was performed using the probe corrected Titan operated at 200 kV with a beam current of 200 pA and a convergence semi-angle of 18.5 mrad. HAADF imaging was performed with an inner collection semi-angle of 54 mrad. EDXS spectrum images of size 512 × 512 pixels were acquired using the Titan's Super-X four

silicon drift detector system with a 30 us dwell time and a total acquisition time of ~5 minutes. EDXS data was acquired and processed using Bruker Esprit software with quantification performed using the Cliff-Lorimer approach without absorption correction. STEM images were acquired using FEI TIA software and processed using the Image-J software.

X-Ray powder diffraction (XRD)

X-ray diffraction (XRD) measurements were carried out on nanoparticles using a Bruker AXS D8 diffractometer. The XRD samples were prepared by pipetting a highly concentrated nanoparticle solution in toluene drop-wise on a small piece of glass slide with a surface area of 10 mm x 10 mm until an estimated 1mm thick solid layer was formed.

Absorption spectroscopy

The absorption spectroscopy measurements for the colloidal HgTe quantum dots were obtained using a Hitachi U-4100 spectrophotometer.

Photoluminescence spectroscopy (PL)

The emission spectra of the colloidal HgTe quantum dots, in toluene, were obtained using 808 nm excitation with the luminescence dispersed in a Bentham TMc300 monochromator and detected using a Newport InGaAs 818-IG detector. The quantum efficiency was obtained using an integrating sphere following the method of de Mello *et al*. [1].

Figures

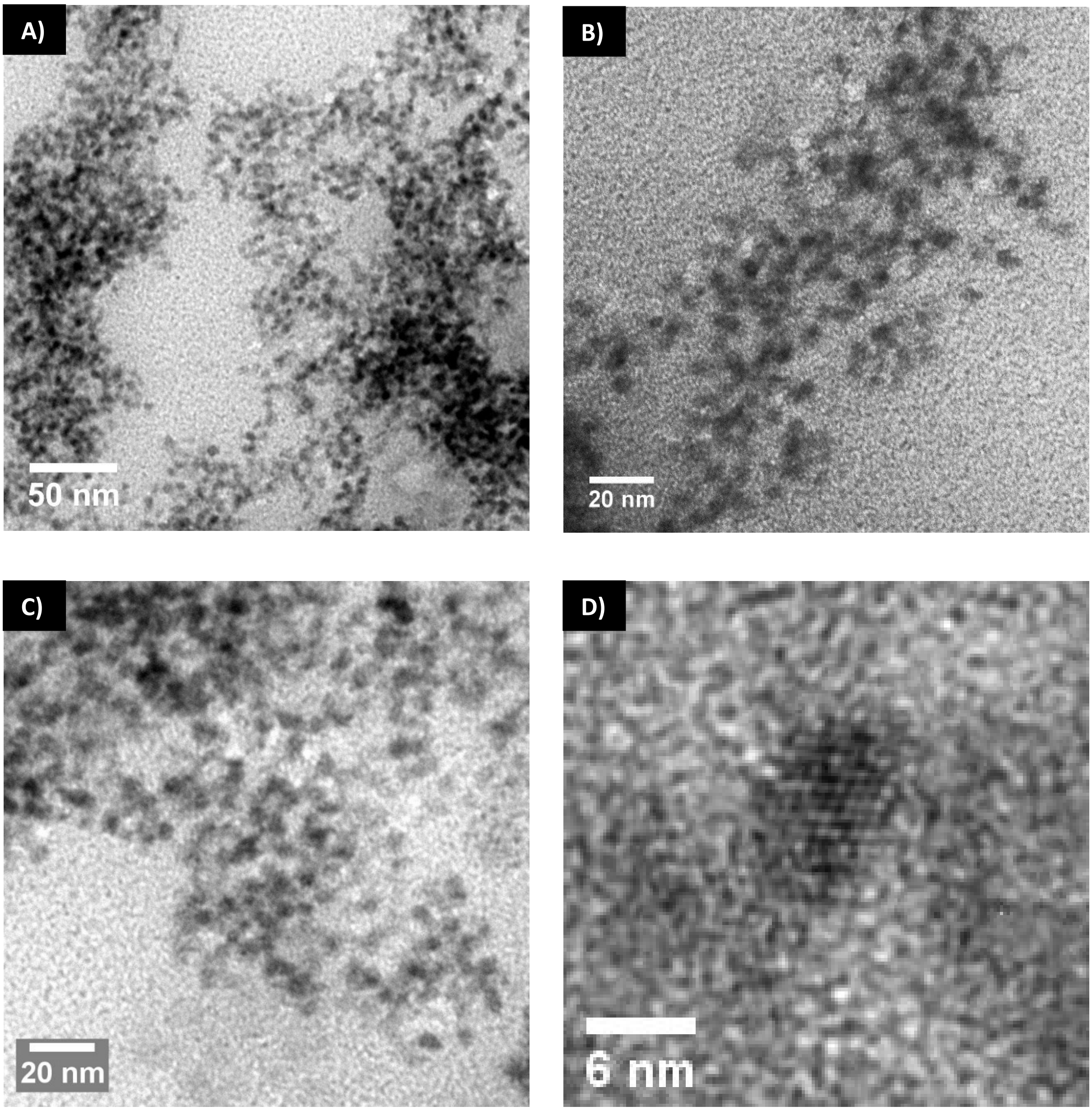

Supporting information figure 1 - TEM images of the HgTe nanoparticles synthesised at room temperature with injection/freezing.

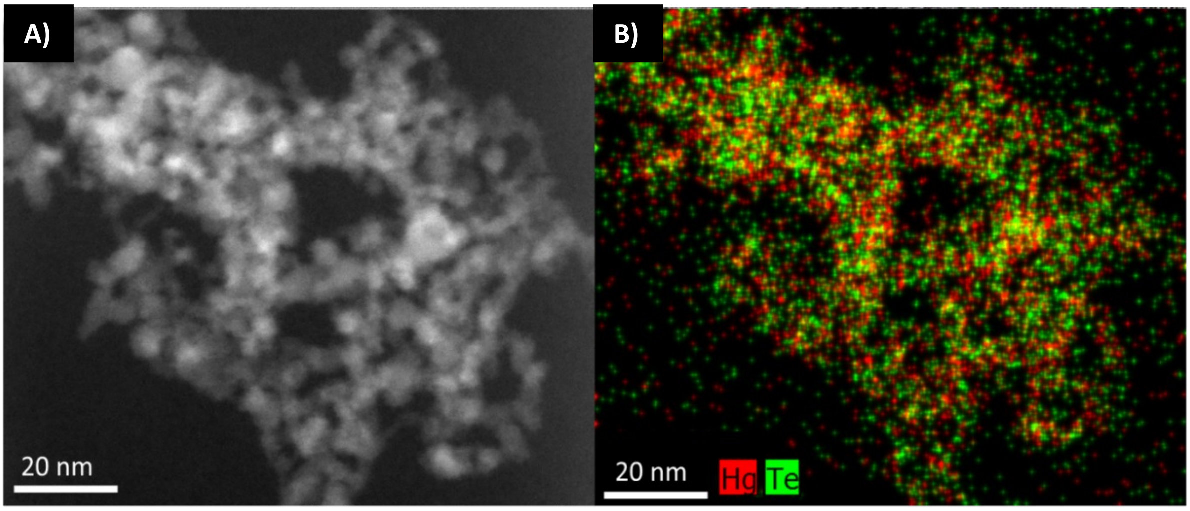

Supporting information figure 2 - STEM A) High-angle annular dark-field imaging (HAADF) STEM image of HgTe QDs; B) STEM-EDX elemental map of the same area showing correlated distributions of Hg and Te.

References

[1] J. C. de Mello, H. F. Wittmann and R. H. Friend, *Adv. Mater.,* 1997, **9,** 230.
